# A novel carboxamide bromodomain inhibitor attenuates osteoarthritis via epigenetic repression of NF-κB and MAPK signaling

**DOI:** 10.3389/fimmu.2025.1633334

**Published:** 2025-07-31

**Authors:** Hyemi Lee, Seong Jae Han, Subin Ok, Kwang Min Lee, Seungseok Choi, Injoo Yoon, Somang Choi, Jina Kim, Serim Ryu, Min-Hee Son, In-Hyun Lee, Chanmi Cho, Siyoung Yang

**Affiliations:** ^1^ Department of Biological Sciences, Sungkyunkwan University, Suwon, Republic of Korea; ^2^ CentralBio Co., Ltd., Incheon, Republic of Korea; ^3^ Department of Life Science and Environmental Biochemistry, and Life and Industry Convergence Research Institute, Pusan National University, Miryang, Republic of Korea; ^4^ New Drug Development Center, Daegu-Gyeongbuk Medical Innovation Foundation, Daegu, Republic of Korea; ^5^ Benobio Co., Ltd, Seongnam, Republic of Korea

**Keywords:** bromodomain, arthritis, carboxamide derivative, mitogen-activated protein kinase, NF-κB

## Abstract

Bromodomains are epigenetic readers that modulate gene expression linked to inflammation and cartilage degeneration. Emerging evidence suggests their dysregulation plays a pivotal role in osteoarthritis (OA) pathogenesis, making them promising therapeutic targets. We evaluated the therapeutic efficacy of a novel carboxamide derivative bromodomain inhibitor (NCD) as a potentially safer alternative for preventing OA progression. The inhibitory effects of NCD were assessed through both *in vitro* and *in vivo* models. *In vitro*, mouse primary chondrocytes were stimulated with IL-1β, and the effects of NCD treatment were analyzed using reverse transcription-polymerase chain reaction (RT-PCR) and western blotting. *In vivo*, destabilization of the medial meniscus (DMM) surgery was performed in 12-week-old male C57BL/6 mice, followed by either oral administration or intra-articular (IA) NCD injection. Cartilage integrity was assessed by histology. We analyzed changes in the NF-κB and mitogen-activated protein kinase (MAPK) signaling pathways to elucidate the mechanism of NCD. NCD treatment significantly suppressed IL-1β-induced expression of matrix metalloproteinases (Mmp3 and Mmp13) and cyclooxygenase-2 (Cox2) in mouse chondrocytes. In the DMM mouse model, both oral IA administration of NCD alleviated OA-related cartilage destruction. Mechanistically, NCD inhibited IκB degradation and reduced Erk and Jnk phosphorylation, indicating suppression of the NF-κB and MAPK signaling pathways. This study demonstrates that targeting bromodomains with a novel carboxamide-based inhibitor effectively attenuates OA cartilage destruction by suppressing these signaling pathways. These findings support the therapeutic potential of epigenetic modulation in mitigating OA pathogenesis.

## 1 Introduction

Osteoarthritis (OA) is the most common degenerative joint disease, which exerts a high socioeconomic impact (1). OA causes the destruction of cartilage, subchondral bone sclerosis, and synovial inflammation (2). Additionally, several OA-associated risk factors, including aging, sex, and obesity, negatively affect multiple physiological processes, ultimately leading to joint destruction. These factors promote cell death, impacting the repair of cracks in the damaged matrix and inflammatory conditions, thus inducing OA progression by creating an imbalance in joints (3, 4). OA involves cartilage degeneration, which is fundamentally induced by the disruption of cartilage homeostasis (5). In articular chondrocytes, the molecular mechanisms of inflammation and cartilage destruction are regulated by the expression of catabolic factors, such as metalloproteinases (MMPs) and cyclooxygenase-2 (Cox2) (6). Mechanical stress and pro-inflammatory cytokines, such as IL-1β, can induce MMP and Cox2 expression, leading to cartilage destruction and OA development (7, 8).

IL-1β modulates the catabolism of chondrocytes by NF-κB signaling, which causes OA (9, 10). NF-κB, a transcription factor, significantly modulates biological processes, such as inflammation, cell differentiation, and cell proliferation (11, 12). Activated NF-κB signaling regulates the expression of MMPs and Cox2, which have been associated with OA progression via the downstream derivation of catabolic factors in chondrocytes (13, 14). Moreover, IL-1β is involved in OA progression by activating mitogen-activated protein kinase (MAPK) subtypes (extracellular signal-regulated kinases [Erk1/2, p38, and Jnk]) in chondrocytes and inducing the expression of MMPs and Cox2 (15, 16). Hence, OA is associated with NF-κB and MAPK signaling, resulting in the expression of catabolic factors in articular chondrocytes.

Among the many MMP isotypes, Mmp3 and Mmp13 are mainly involved in degrading the chondrocyte matrix. Mmp3 and Mmp13 exhibit aggrecanase and collagenase activities (17, 18) and break down extracellular matrix (ECM) components. Cox2 is mainly involved in joint inflammation, activates Mmp3 and Mmp13, and eventually promotes ECM degradation during OA development (19, 20). Therefore, the ECM degrades with increasing levels of Cox2, Mmp3, and Mmp13 during OA development, leading to critical symptoms.

Recent studies have highlighted the role of epigenetic regulation in OA, where histone modifications and chromatin remodeling influence the transcriptional activation of pro-inflammatory and catabolic genes in chondrocytes (21). Among epigenetic regulators, bromodomain-containing proteins serve as “readers” of acetylated histones, facilitating the transcription of inflammation- and matrix degradation-related genes. JQ1 inhibits bromodomain and extra-terminal domain (BET) proteins, and recent reports indicate that it impedes the progression of various cancers and rheumatoid arthritis (22). Additionally, it inhibits OA development by suppressing the NF-κB signaling pathway (23). However, JQ1 is considered unsafe at pharmacological concentrations, and several side effects have been reported (24). To address the limitations of existing BET inhibitors, we synthesized a novel carboxamide-derived BET inhibitor, which we refer to as the new carboxamide derivative (NCD). NCD shows improved pharmacological safety and dual-delivery capabilities, including both via oral and intra-articular (IA) administration. This compound was designed to retain potent anti-inflammatory effects while minimizing off-target toxicity.

Currently, no disease-modifying therapy is available to prevent the progression of OA. Existing disease management strategies focus on pain control and functional improvement, avoiding treatment toxicity (1). In the present study, we aim to estimate the potential of a new carboxamide derivative (NCD), which is a novel BET inhibitor, for suppressing OA progression. We analyzed its effect on Mmp3, Mmp13, and Cox2 expression, as well as explored its correlation with NF-κB and MAPK signaling pathways through *in vitro* and *in vivo* experiments. Moreover, we discussed the use of NCD in the development of new drugs for OA treatment.

## 2 Materials and methods

### 2.1 Screening of DNA-encoded chemical library with bromodomains from BRD2, BRD3, and BRD4

Novel bromodomain and extra-terminal domain inhibitors (BETi) were identified through screening of a DNA-encoded chemical library (DEL; WuXi AppTec, Shanghai, China) using the BD1 and BD2 domains from BRD2, BRD3, and BRD4 proteins. These bromodomain proteins were produced by amplifying the corresponding gene segments, inserting them into pET28a expression vectors (Novagen, Northumberland, UK), and transforming *Escherichia coli* BL21(DE3) cells (Novagen) with the recombinant plasmids. Protein expression was induced with 0.2 mM isopropyl β-D-thiogalactopyranoside (IPTG; Sigma Aldrich, St. Louis, MO, USA) at 18°C for 16 h. The bacterial cells were harvested, resuspended in lysis buffer (50 mM Tris, pH 8.2; 300 mM NaCl; 20 mM imidazole), and lysed by sonication. The lysates were centrifuged at 1,550 × *g* for 1 h at 4°C, and the resulting supernatants were applied to nickel-affinity HisTrap HP columns (GE Healthcare, Chicago, IL, USA). The bound proteins were eluted with 50 mM Tris buffer (pH 8.2) containing 300 mM NaCl and 500 mM imidazole.

### 2.2 Time-resolved fluorescence resonance energy transfer assay

TR-FRET assays were conducted using kits specific for BRD2 (BD1 + BD2), BRD3 (BD1 + BD2), and BRD4 (BD1 + BD2) obtained from BPS Bioscience (San Diego, CA, USA). Bromodomain ligands were diluted in water, and master mixes were prepared by combining the diluted ligands with 1× BRD Homogeneous Assay Buffer. Thawed BRD proteins were also diluted with the same buffer. For each assay, 1.5 μL of the master mix was dispensed into wells of a microplate (Labcyte, San Jose, CA, USA), followed by the addition of 5 μL of diluted BRD protein to initiate the reaction. Plates were incubated at 20–22°C for 30–60 min. Subsequently, glutathione (GSH) acceptor beads and streptavidin-conjugated donor beads (both from PerkinElmer, Waltham, MA, USA) were diluted in 1× BRD Homogeneous Detection Buffer 1. Next, 10 μL of the acceptor bead solution was added to each well and incubated at 18°C for 30 min, followed by the addition of 10 μL of donor bead solution and a further incubation at 18°C for 15–30 min. Alpha particle counts were measured using an EnVision 2105 multimode plate reader (PerkinElmer).

### 2.3 Binding kinetics assay

Binding kinetics were assessed using the Octet^®^ R2 system (Sartorius, Göttingen, Germany). Superstreptavidin biosensors were loaded with biotinylated BRD2 BD1, BRD2 BD2, BRD3 BD1, BRD3 BD2, BRD4 BD1, BRD4 BD2, or biotin alone (as a negative control) in PBST buffer (phosphate-buffered saline containing 0.02% Tween 20 and 5% DMSO). The sensors were then dipped into compound solutions for 10 min. To evaluate association kinetics (kon), each sensor was exposed to serial dilutions of the protein for 180 s, followed by dipping them into PBST buffer to assess dissociation kinetics (koff). For control, a separate sensor loaded with the compound was dipped into PBST alone. Competition binding assays were performed by exposing the compound-loaded sensor to mixtures of proteins and test compounds.

### 2.4 Mice

All *in vivo* experiments included in this study were approved by the Animal Care and Use Committee of Sungkyunkwan University (SKKUIACUC2023-07-52-1) and were conducted following the 8th edition of the Guide for the Care and Use of Laboratory Animals issued by the National Institutes of Health. Five-day-old Institute of Cancer Research (ICR) and C57BL/6 mice were purchased from DBL Co., Ltd. (Chungbuk, Korea). Primary cultures of articular chondrocytes isolated from 5-d-old ICR mice were established. A destabilization of the medial meniscus (DMM) mouse model was created using C57BL/6 mice. C57BL/6J male mice weighing 18–20 g (age, 12 weeks) were exposed to a 12/12-h light-dark cycle, while providing water and food regularly. C57BL/6J male mice were housed at 23°C.

### 2.5 Primary culture of articular chondrocytes and viability analysis

For *in vitro* experiments, cells were plated using a randomized layout across multi-well plates to avoid positional effects and ensure unbiased treatment assignment. Articular chondrocytes were acquired from the femoral and tibial plateaus of 5-d-old postnatal ICR mice. The mouse cartilage tissue was digested using 0.2% collagenase type II (Sigma–Aldrich, MO, USA). Mouse chondrocytes were seeded in 96-well dishes (9 × 10^3^ cells/well) and incubated for 48 h, followed by treatment with the NCD and JQ1. Articular chondrocytes primary cultures were incubated in Dulbecco’s modified Eagle’s medium supplemented with 10% fetal calf serum (Capricon, Ebsdorfergrund, Germany) and 1% penicillin-streptomycin (Capricon, Ebsdorfergrund, Germany). NCD treatment was performed using different concentrations (10, 50, and 100 μM) of NCD for 12 h. Cell viability was determined using the culture medium through the lactate dehydrogenase (LDH) activity assay using the LDH Colorimetric Assay Kit (BioVision Inc, Milpitas, USA) (25, 26). The signal was measured at 495 nm using a SYNERGY H1 Microplate Reader (Biotek, Winooski, USA) (27).

### 2.6 Reagents and treatment

NCD was supplied by Benobio Co (Gyeonggi-do, Korea), and IL‐1β was purchased from GenScript (Piscataway, NJ, USA). NCDs were dissolved in phosphate-buffered saline (PBS) for oral administration or IA injection. Mouse articular chondrocytes were treated with IL‐1β (1 ng/mL) to create an *in vitro* OA condition and co-treated with NCD (10, 50, and 100 μM) or JQ1 (200 nM, positive control) for 12 h before harvesting.

### 2.7 Quantitative reverse transcription-polymerase chain reaction

Total RNA was extracted from mouse articular chondrocytes using TRIzol (Molecular Research Center Inc., Cincinnati, OH, USA) and the primers used were for the MMPs, glyceraldehyde 3-phosphate dehydrogenase (GAPDH), and Cox2. Primers utilized were as follows: mouse *Mmp3* (5′-TCCTG ATGTT GGTGG CTTCA G-3′ and 5′-TGTCT TGGCA AATCC GGTGT A-3′); mouse *Mmp13* (5′-TGATG GACCT TCTGG TCTTC TGG-3′ and 5′-CATCCA CATGG TTGGG AAGT TCT-3′); mouse *Cox2* (5′-GGTCT GGTGC CTGGT CTGAT GAT-3′ and 5′-GTCCT TTCAA GGAGA ATGGT GC-3′); mouse *Gapdh* (5′-TCACT GCCAC CCAGA AGAC-3′ and 5′-TGTAG GCCAT GAGGT CCAC-3′). Gene transcript expression levels were normalized against that of *Gapdh* by qRT-PCR using SYBR premix ExTaq (Takara Bio, Shiga, Japan). Results were expressed as fold-changes relative to the expression in the control group (20).

### 2.8 Protein isolation and western blotting

The total protein was extracted from the primary cultured chondrocytes using a lysis buffer, containing 150 mM NaCl, 1% NP-40, 50 mM Tris/HCl (pH 8.0), 0.2% sodium dodecyl sulfate, and 5 mM NaF, supplemented with protease and phosphatase inhibitor mixture (Roche, Madison, WI, USA). Proteins were separated using sodium dodecyl sulfate-polyacrylamide gel electrophoresis, and western blot analysis was performed using the following antibodies: goat anti‐Cox2 (sc‐1745; Santa Cruz Biotechnology, Dallas, TX, USA), mouse anti-Erk1/2 (610408; Becton Dickinson, NJ, USA), mouse anti-IκB [9242; Cell Signaling Technology (CST), MA, USA], mouse anti-p65 (8242; CST), mouse anti-phospho-p65 (3033; CST), mouse anti-p38 (#9212; CST), mouse anti-pp38 (#9215S; CST), mouse anti-c-Jun N-terminal kinase (Jnk) (#9252S; CST), mouse anti-pJnk (#9251S; CST), and mouse anti-pErk (#9101S; CST). Each signal was visualized using a SuperSignal West Dura Kit (Thermo Fisher Scientific, Waltham, MA, USA). Density analysis (AlphaEase FC 4.0; Alpha Innotech, CA, USA) was performed to calculate the relevant band intensities. Extracellular signal-regulated kinase (Erk) was used as the loading control (28).

### 2.9 Prostaglandin E2 assays

PGE_2_ expression was determined by enzyme-linked immunosorbent assay (ELISA) using a PGE_2_ Immunoassay Kit (R&D Systems, Minneapolis, MN, USA). The levels of PGE_2_ in the conditioned medium of articular chondrocytes were determined. Collagenase activity was measured using a VICTOR X3 Microplate Reader (PerkinElmer, Waltham, MA, USA) at the excitation/emission wavelengths of 490/530 nm following the protocol provided by the manufacturer.

### 2.10 Oral administration or IA injection in DMM mice

All experiments were independently performed with at least three biological replicates to ensure reproducibility. For *in vivo* studies, animals were randomly assigned to treatment or control groups. To create a DMM-induced OA model, DMM surgery was performed on 12-week-old male C57BL/6J mice, following a previously reported protocol (26). Subsequently, NCD was either orally administered or injected in mice; the NCD (50 mg/kg) was orally administered on alternate days 4 weeks after DMM, whereas IA knee injection of NCD (10 μg) was administered once a week after 4 weeks. The mouse knee joint was processed for histological analysis 10 weeks after surgery. All experiments were independently performed with at least three biological replicates to ensure reproducibility. For *in vivo* studies, animals were randomly assigned to treatment or control groups. For *in vitro* experiments, cells were plated using a randomized layout across multi-well plates to avoid positional effects and ensure unbiased treatment assignment

### 2.11 Evaluation of cartilage destruction and immunohistochemical analysis

All histological samples were prepared independently by two researchers. Cartilage destruction was evaluated based on Safranin O staining and scored using the OA Research Association International (OARSI) grading system (29). Additionally, all histological samples were analyzed through osteophyte maturity scoring and subchondral bone plate (SBP) thickness. Osteophyte maturity was scored as described previously (30). Briefly, the scoring was as follows: 0 = none, 1 = predominantly cartilaginous, 2 = mixed cartilage and bone with active vascular invasion and endochondral ossification, 3 = predominantly bone. SBP thickness was measured to analyze subchondral bone sclerosis. All experiments were independently performed over at least three replicates. A perpendicular line was drawn from the cartilage–bone interface to the inferior border of the compact subchondral bone using NIS-Elements software (Nikon), as described previously (25). For each sample, measurements were performed on serial sections, and the mean value was calculated for analysis. The samples were dehydrated by treating with 0.5 M ethylenediamine tetraacetic acid (pH 8.0) for 2 weeks. Next, mouse knee joint samples were fixed using 4% paraformaldehyde and embedded in paraffin. The paraffin-embedded samples were cut into 5-μm sections and fixed on glass slides. The tissue sections were then hydrated using a graded ethanol series, which removed paraffin and xylene. Mouse knee joint sections were immunohistochemically analyzed using anti-Mmp3 (ab52915, Abcam, Cambridge, UK), anti-Mmp13 (ab51072, Abcam), and anti-Cox2 (66351-I-Ig, ProteinTech, Rosemont, ILllinois, USA).

### 2.12 PK analysis of NCD

SD rats (male, 6 weeks old) were obtained from Orient Bio (Seoul, Korea) and housed under pathogen-free conditions. All animal procedures were conducted in accordance with the guidelines of the Animal Care Facility at Biotoxtech Co., Ltd. (Chungcheongbuk-do, Korea). NCD was administered intravenously (IV) to SD rats at doses of 5, 10, or 20 mg/kg to evaluate its pharmacokinetics. Blood samples were collected from the jugular vein at various time points, mixed with 3.8% (w/v) sodium citrate, and centrifuged at 8,000 × *g* for 30 min to obtain plasma. The plasma samples were then mixed with acetonitrile (33%, v/v), vortexed for 5 min, and centrifuged at 13,000 × *g* for 10 min. The concentration of NCD in plasma was determined using high-performance liquid chromatography (HPLC; Shimadzu, Japan) equipped with a UV detector. Pharmacokinetic parameters were calculated using Microsoft Excel (31).

### 2.13 Statistical analysis

The data are presented as mean ± standard error of mean (SEM). Each experiment was performed at least three times. One-way analysis of variance with the Bonferroni *post hoc* test was performed for data analysis. The PRISM 9 software was used for statistical analysis. The cut-off for statistical significance was set at P ≤ 0.05.

## 3 Results

### 3.1 NCD directly bind to BRD2 without any toxic effects

**Figure 1:**
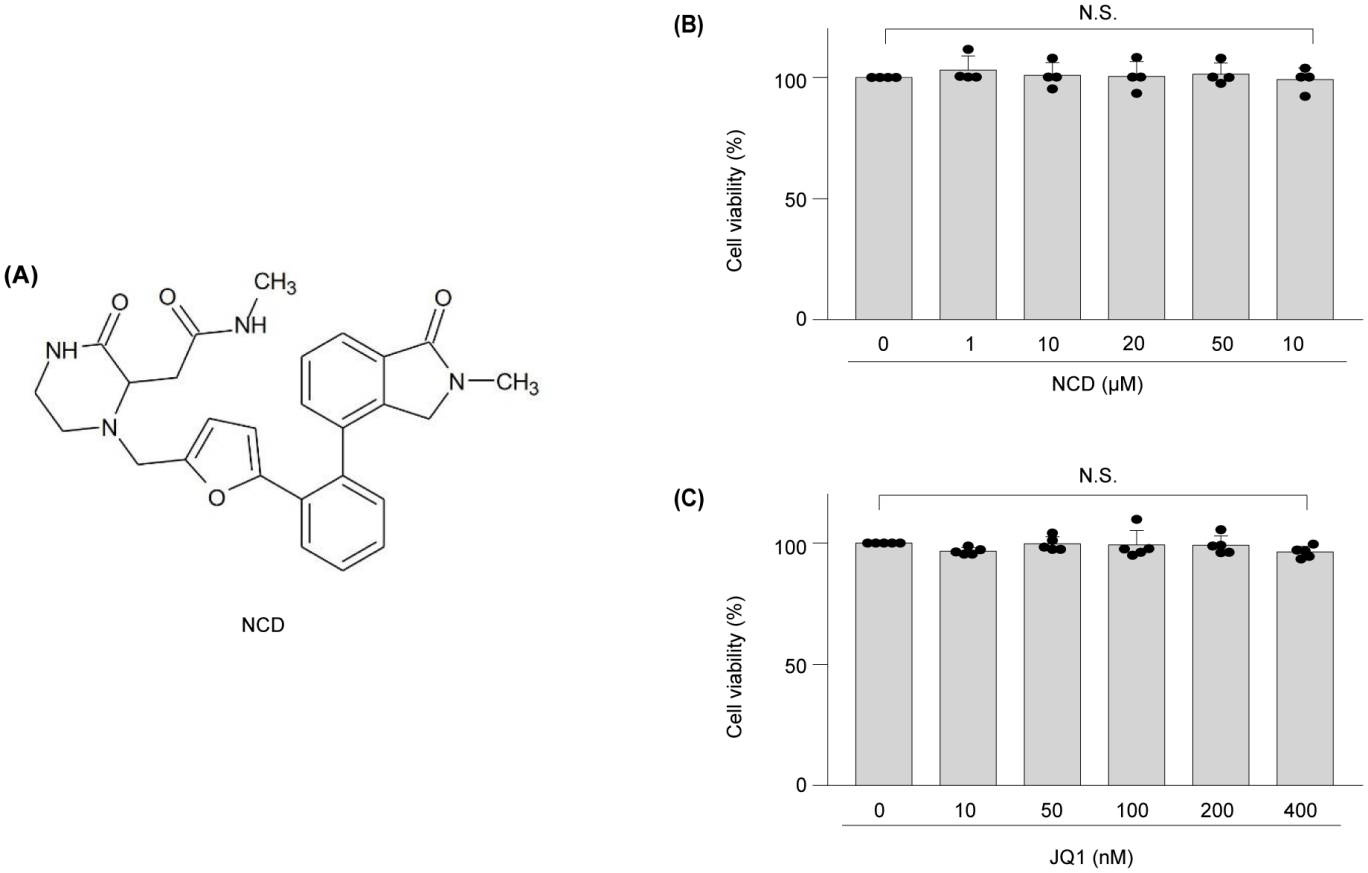
Structure of NCD, and toxicity of NCD or JQ1 to mouse articular chondrocytes analyzed *in vitro*. **(A)** Structure of NCD. **(B, C)** Viability of chondrocytes was measured via a lactate dehydrogenase (LDH) assay after treatment with NCD **(B)** or JQ1 **(C)** at different concentrations for 12 h Data were analyzed via one‐way analysis of variance with Bonferroni’s test and expressed as mean ± standard error of mean (SEM); N.S., not significant.

**Figure 2:**
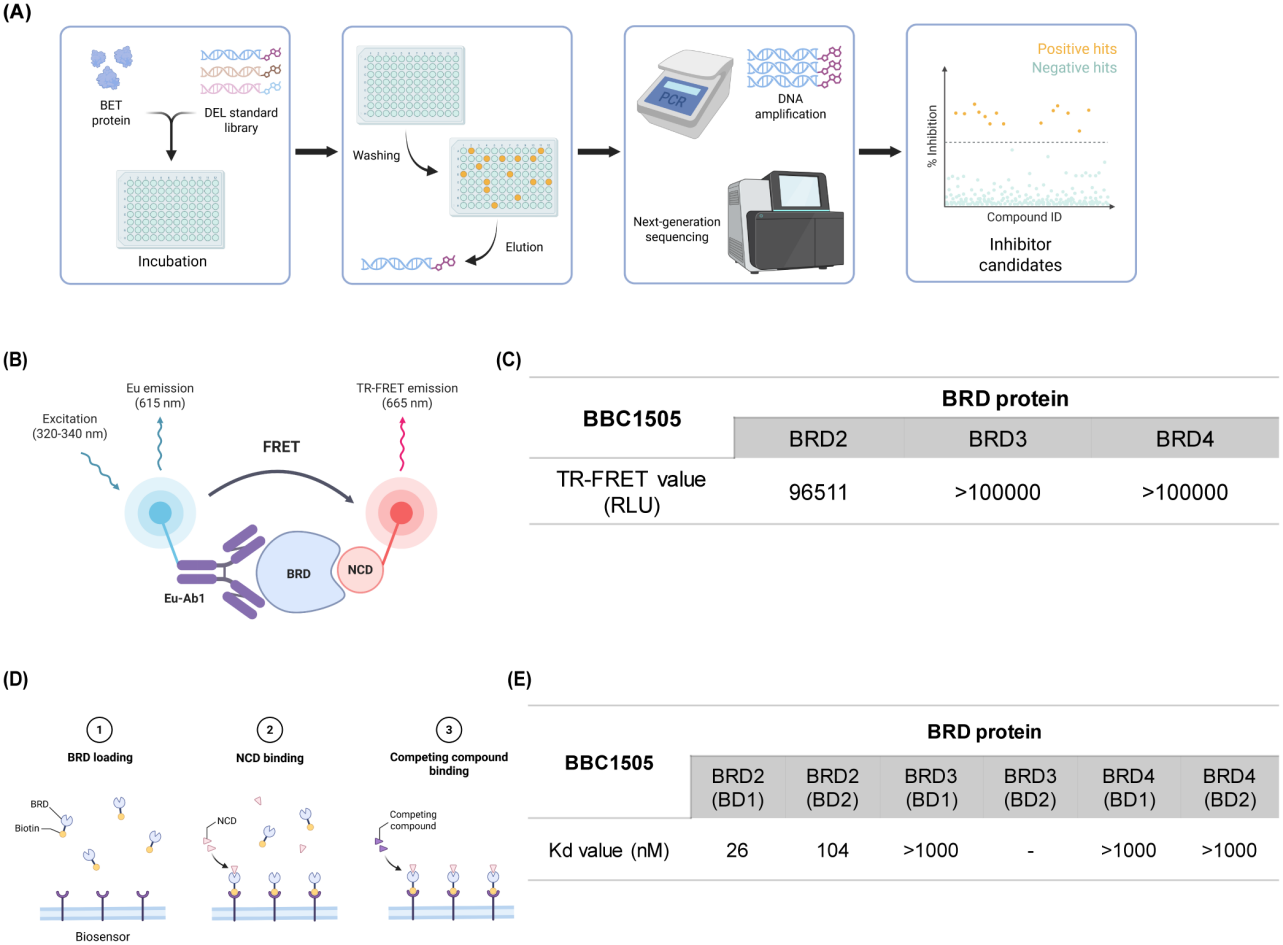
NCD selectively binds to BRD2 **(A)** Schematic representation of the DNA-encoded library (DEL) screening process. Purified target proteins were incubated with the DEL standard library. DNA-tagged compounds that bound to the target were isolated, amplified by PCR, and identified using next-generation sequencing. **(B, C)** Schematic and quantitative results of the TR-FRET assay using NCD. A lower TR-FRET signal indicates stronger binding to the bromodomain (BD), as it prevents the probe from binding to the ligand. NCD exhibited selective binding, with reduced TR-FRET signals observed for BRD2, indicating high affinity. **(D, E)** Schematic and results of the binding kinetics assay using a biosensor. Binding affinities (KD values) of NCD for the bromodomains of BRD2–4 were measured. Lower KD values indicate stronger binding affinity, with NCD showing the highest affinity for BRD2.

The NCD used was N-methyl-2-(1-((5-(2-(2-methyl-1-oxoisoindolin-4-yl)phenyl)furan-2-yl)methyl)-3-oxopiperazin-2-yl)acetamide (**Figure 1A**). To ensure its suitability for subsequent *in vitro* experiments, we first evaluated the cytotoxicity of NCD in primary articular chondrocytes. Cell viability was assessed following treatment with increasing concentrations of NCD (0, 10, 20, 50, and 100 µM) and compared the viability of cells with those treated using the well-characterized BET inhibitor JQ1, used here as a reference compound. The results revealed that NCD exhibited no significant cytotoxic effects across all tested concentrations, with cell viability remaining comparable to that of the untreated control group (**Figure 1B**). In contrast, JQ1 treatment led to a marked decrease in cell viability, even at lower concentrations (**Figure 1C**). These findings confirm the favorable safety profile of NCD in articular chondrocytes and support its use for further mechanistic and functional studies. Accordingly, we selected concentrations of 0, 10, 50, and 100 µM for all subsequent *in vitro* assays to assess the biological and pharmacological effects of NCD. To support the target specificity of NCD as a BET inhibitor, we performed a DEL screening using the WUXI platform (**Figure 2A**). This screening identified NCD as a candidate compound with high affinity for BRD2. To validate this interaction, we conducted a TR-FRET assay, which demonstrated that NCD showed markedly lower TR-FRET signals for BRD2 compared to those of BRD3 and BRD4, indicating stronger binding affinity (**Figures 2B, C**). Furthermore, biosensor-based binding kinetics assays revealed that NCD exhibited the lowest dissociation constant (KD) for BRD2, confirming its selective and high-affinity interaction (**Figures 2D, E**). Together, these findings establish the molecular selectivity of NCD for BRD2 among BET bromodomains.

### 3.2 NCD inhibited the IL-1β-induced expression of MMPs and Cox2 in chondrocytes

**Figure 3:**
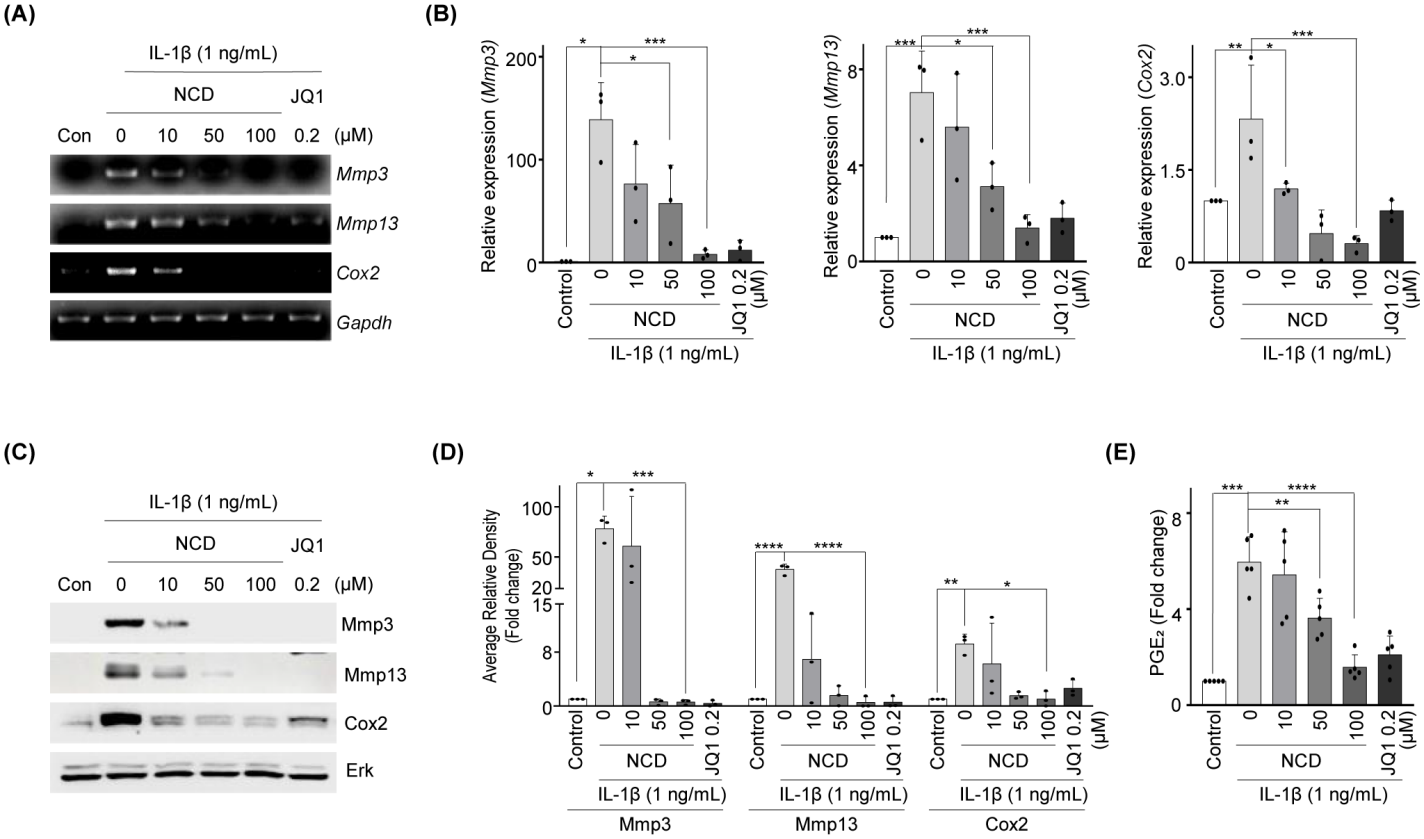
NCD reduces the expression of MMPs and Cox2 and inhibits the IL-1β-induced production of PGE_2_ in mice chondrocytes. Chondrocytes stimulated using IL‐1β (1 ng/mL) were treated with different concentrations of NCD (0, 10, 50, or 100 µM); JQ-1 (200 µM) served as a positive control. **(A, B)** The mRNA expression of Mmp3 and Cox2 was analyzed using RT‐PCR and qRT-PCR (n = 3). **(C, D)** The protein level of Mmp3, Mmp13, and Cox2 was determined using western blotting and densitometry (n = 3). **(E)** PGE_2_ assay was performed with mouse articular chondrocytes treated with different concentrations of NCD (0, 10, 50, and 100 µM) after IL-1β stimulation (1 ng/mL) (n = 5). Glyceraldehyde 3-phosphate dehydrogenase (GAPDH) and extracellular signal-regulated kinase (Erk) were used as loading controls. All data were analyzed using one‐way analysis of variance with Bonferroni’s test and expressed as mean ± standard error of mean (SEM); *P < 0.05, **P < 0.01, ***P < 0.001, and ****P < 0.0001 compared to the control group.

IL-1β elevated the expression of matrix-degrading enzymes Mmp3 and Mmp13, as well as the inflammatory mediator Cox2, consistent with previous reports implicating these factors in OA-related cartilage destruction (32, 33). To evaluate whether NCD could counteract these IL-1β-induced catabolic responses, we conducted qRT-PCR analyses following co-treatment with NCD. The results revealed a marked, concentration-dependent suppression of Mmp3, Mmp13, and Cox2 mRNA expression after 12 h of NCD exposure. Notably, both mRNA and protein levels of Cox2 were substantially reduced at NCD concentrations of 50 and 100 µM, indicating a robust anti-inflammatory effect of the compound (**Figures 3A–D**). Furthermore, ELISA quantification of PGE_2_—a downstream product of Cox2 enzymatic activity—showed that NCD significantly decreased PGE_2_ production in IL-1β-treated chondrocytes in a dose-dependent manner (**Figure 3E**). These findings collectively suggest that NCD effectively suppresses both the transcriptional and translational upregulation of key catabolic and inflammatory mediators induced by IL-1β, thereby highlighting its potential as a disease-modifying therapeutic agent in OA.

### 3.3 Orally administered and injected NCD prevented cartilage destruction in the DMM-induced OA mouse model

**Figure 4:**
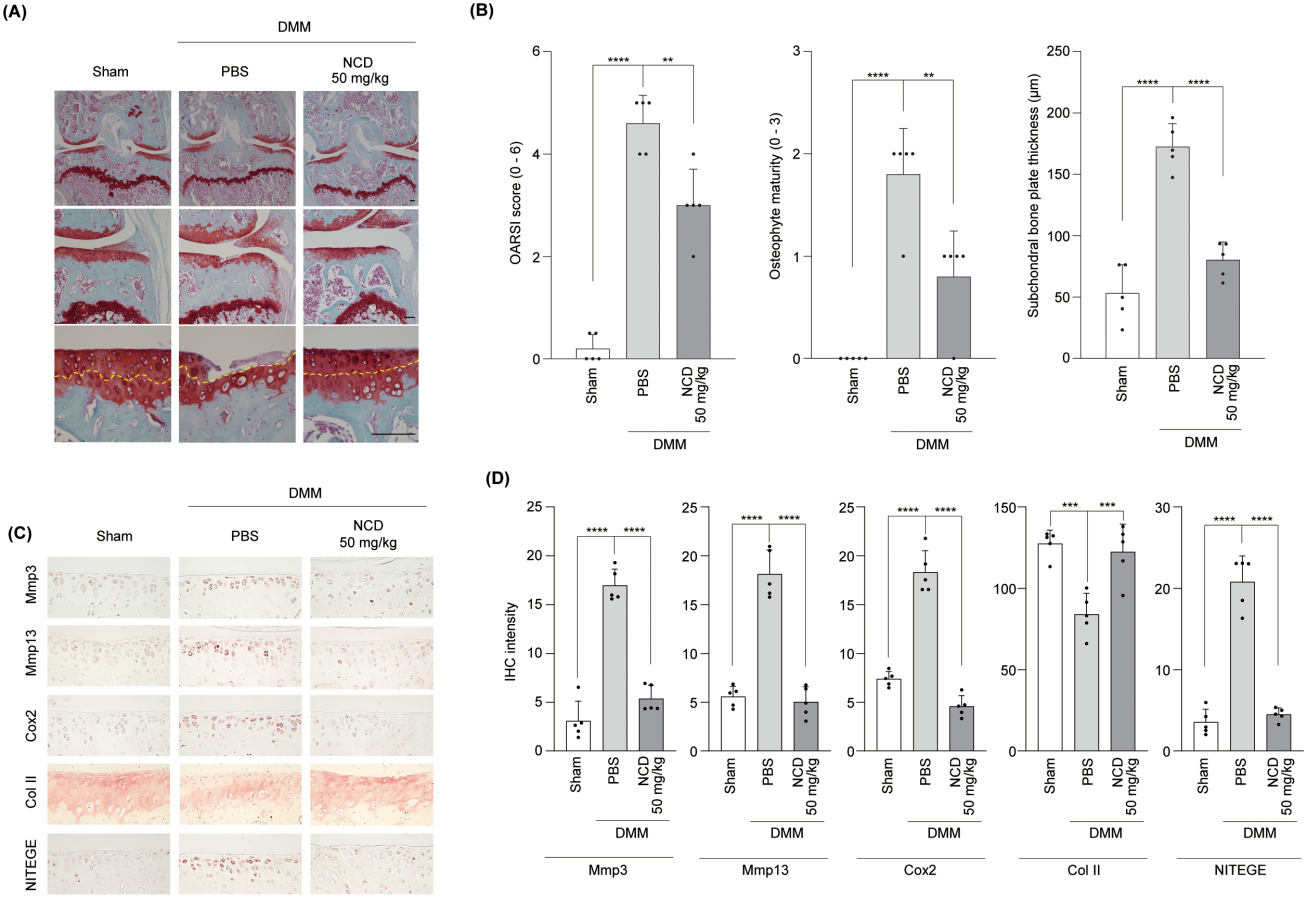
Oral Administration of NCD prevents mouse articular cartilage destruction in OA. NCD was orally administered into DMM mice on alternate days from 4 weeks after surgery. **(A, B)** Cartilage degradation and OA development were analyzed through Safranin O staining **(A)**, OARSI scores (B, left panel), osteophyte maturity **(B)**, middle panel), and SBP thickness **(B)**, right panel) measurements 10 weeks after DMM surgery. The yellow arrows point to the tidemark. **(C, D)** The expression of Mmp3, Mmp13, Cox2, Col II, and NITEGE was determined through immunohistochemical analysis. All images were captured at magnifications of ×40, ×100, and ×400. Statistical analysis was performed using one-way analysis of variance (ANOVA) followed by Bonferroni’s *post hoc* test. Data are presented as mean ± standard error of the mean (SEM) (n = 5). **P < 0.01, ***P < 0.001, and ****P < 0.0001, compared to the control (PBS-treated) group. Scale bar = 100 μm.

To further validate the therapeutic efficacy of NCD *in vivo*, we employed a surgically induced OA model using DMM in mice and assessed the outcomes following both oral and IA administration. Oral administration significantly attenuated cartilage degeneration, as evidenced by histological analysis showing preservation of cartilage structure and a concomitant decrease in the OARSI score, osteophyte maturity, and SBP thickness in NCD-treated mice relative to vehicle-treated controls (**Figures 4A, B**), suggesting OA progression was substantially inhibited. Immunohistochemical staining further supported these findings, demonstrating markedly reduced expression levels of matrix-degrading enzymes Mmp3 and Mmp13, as well as the inflammatory mediator Cox2, in the articular cartilage of NCD-administered animals. Additionally, oral NCD treatment restored the presence of type II collagen, a key structural component of healthy cartilage, and significantly reduced levels of NITEGE, a well-established neoepitope marker indicative of aggrecan degradation (34), thereby reflecting an overall improvement in cartilage matrix integrity (**Figures 4C, D**).

**Figure 5:**
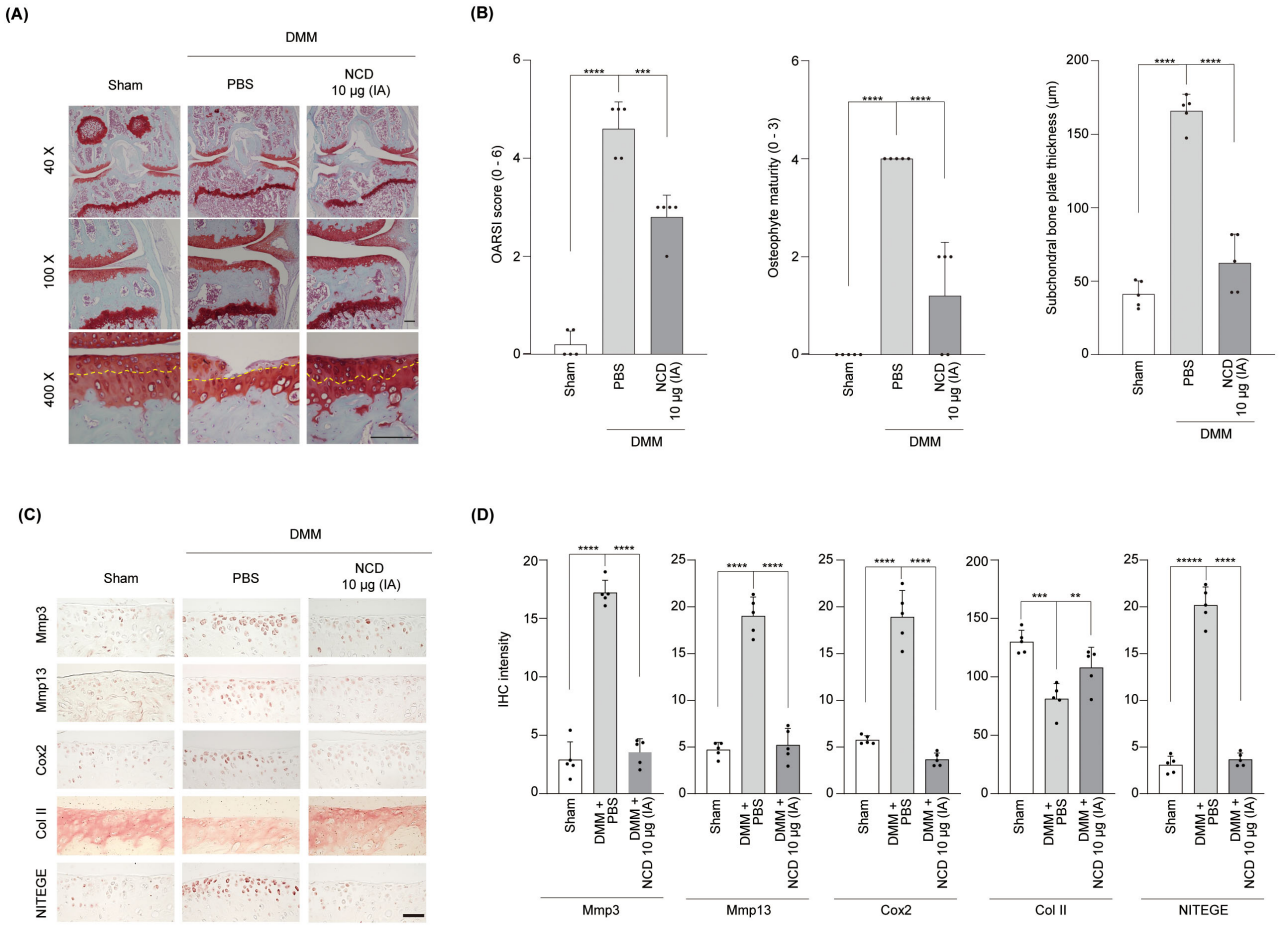
Intra-articular (IA) knee injection of NCD prevents mouse articular cartilage destruction in OA. IA injection of NCD was administered in DMM mice once weekly from 4 weeks after surgery. **(A)** Analysis of cartilage degradation was performed through Safranin O staining. **(B)** Cartilage destruction and OA development were determined using OARSI scores (B, left panel), osteophyte maturity (B, middle panel), and SBP thickness (B, right panel) at 10 weeks following surgery to destabilize the medial meniscus The yellow arrows point to the tidemark. **(C, D)** Expression of Mmp3, Mmp13, Cox2, Col II, and NITEGE was determined by immunohistochemistry. All images were captured at magnifications of ×40, ×100, and ×400. Statistical analysis was performed using one-way analysis of variance (ANOVA) followed by Bonferroni’s *post hoc* test. Data are presented as mean ± standard error of the mean (SEM) (n = 5). **P < 0.01, ***P < 0.001, and ****P < 0.0001, compared to the control (PBS-treated) group. Scale bar = 100 μm.

To determine whether localized delivery of NCD via IA injection exerts similar protective effects, we next evaluated its impact on DMM-induced OA in a separate cohort of mice. Consistent with the effects observed following oral administration, IA injection effectively prevented cartilage destruction, as shown by improved histological outcomes and a significant reduction in OARSI grade, osteophyte maturity, and SBP thickness compared to PBS-treated controls (**Figures 5A. B**). Immunohistochemical analyses corroborated these observations, revealing lower expression levels of Mmp3, Mmp13, and Cox2 in NCD-injected knees relative to controls. Interestingly, while type II collagen levels were partially preserved, NITEGE expression was also reduced in NCD-treated joints, indicating protection against ECM degradation (**Figures 5C, D**). Collectively, these results suggest that NCD, whether administered by oral or IA routes, exerts potent chondroprotective effects by mitigating inflammatory and catabolic processes in articular cartilage, thereby effectively suppressing the progression of OA without causing detectable organ toxicity *in vivo* (**Supplementary Figures 1**, and **Supplementary Figures 2**).

Although the pharmacokinetics of the oral and intra-articular administration of NCD were not directly assessed in the current study, we analyzed its pharmacokinetic profile following IV injection to establish its systemic exposure characteristics. As shown in **Supplementary Table 1**, pharmacokinetic parameters including C_max_, half-life, and AUC were determined. As the dose was increased from 5 to 20 mg/kg, the C_max_ rose proportionally from 1111.52 ng/mL to 4150.81 ng/mL, indicating a dose-dependent increase in systemic exposure. Similarly, AUC increased from 631.83 to 4526.66 ng·h^−1^mL^−1^, reflecting enhanced overall drug exposure at higher doses. Notably, the half-life of NCD extended from 0.41 to 0.76 h, suggesting a dose-dependent increase in the time required for the drug to be metabolized and eliminated These results support the pharmacokinetic scalability of NCD and provide a basis for future dose optimization and formulation development.

### 3.4 NCD blocked IL-1β-induced activation of NF-κB and MAPK signaling in mouse articular chondrocytes

**Figure 6:**
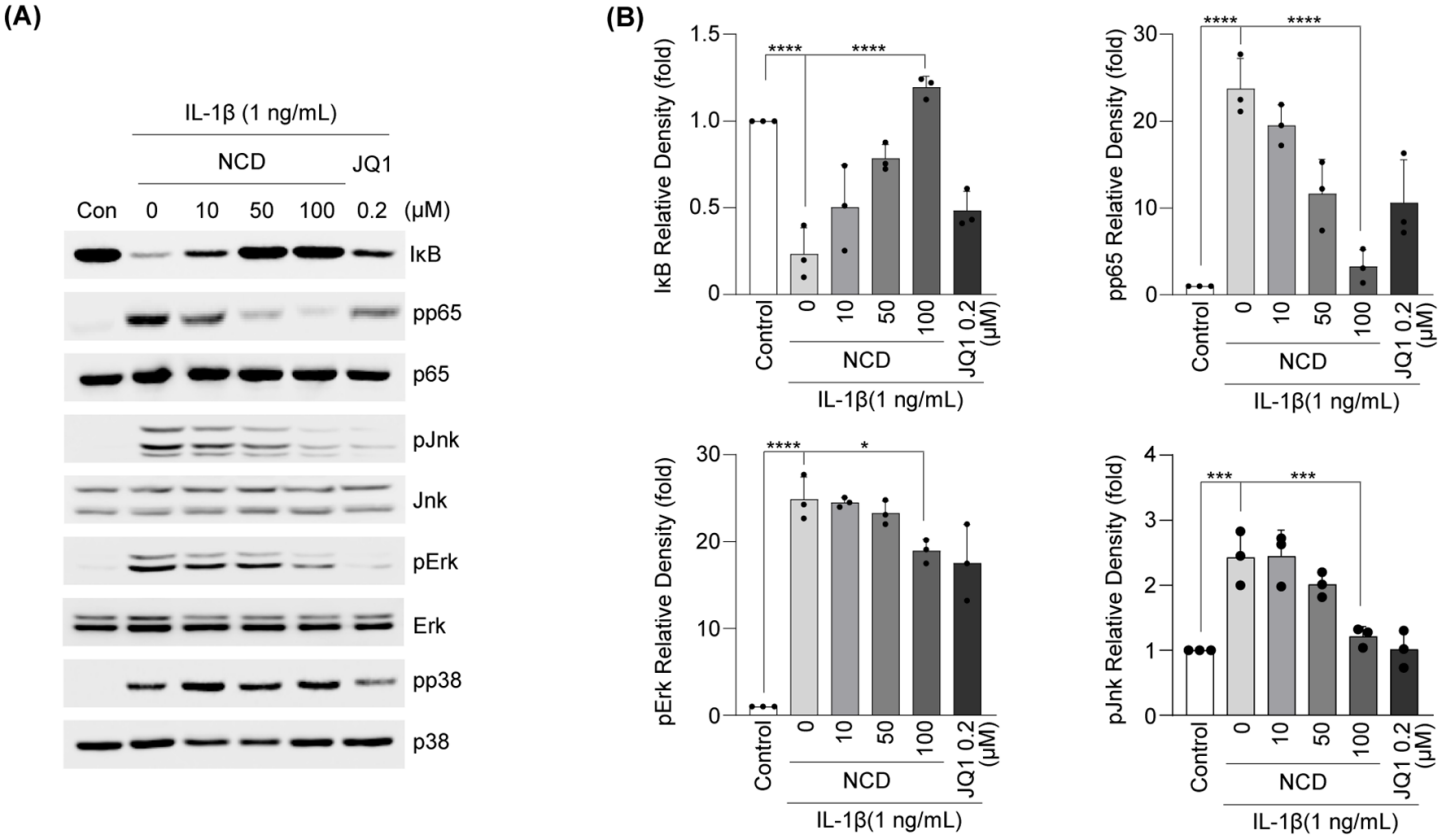
NCD regulates NF-κB and MAPK signaling pathways induced by IL-1β in mouse chondrocytes. Articular chondrocytes were pretreated with NCD at different concentrations (0,10, 50, or 100 μM) for 12 h (n = 3) before being stimulated with IL-1β (1 ng/mL) for 10 min. Levels of phosphorylated Jnk (pJnk), phosphorylated Erk (pErk), and phosphorylated p38 (pp38) proteins were determined using western blotting. **(A, B)** Protein levels of IκB were detected using western blotting **(A)** and densitometry **(B)**. JQ-1 (200 μM) was used as a positive control. All data were analyzed using one‐way analysis of variance with Bonferroni’s test and expressed as mean ± standard error of mean (SEM) (n = 3); *P < 0.05, **P < 0.01, ***P < 0.001, and ****P < 0.0001 compared to the control group.

To elucidate the molecular mechanisms by which NCD exerts its protective effects against OA progression, we investigated its influence on key intermediates of the NF-κB and OA signaling pathways. Western blotting analysis combined with densitometric quantification revealed that NCD treatment effectively prevented the degradation of IκB, an inhibitory molecule that regulates the cytoplasmic retention of NF-κB. Furthermore, NCD significantly suppressed the phosphorylation of the p65 subunit, a critical component of the NF-κB transcriptional complex, thereby implicating a blockade of NF-κB nuclear translocation and downstream gene activation (**Figures 6A, B**).

**Figure 7:**
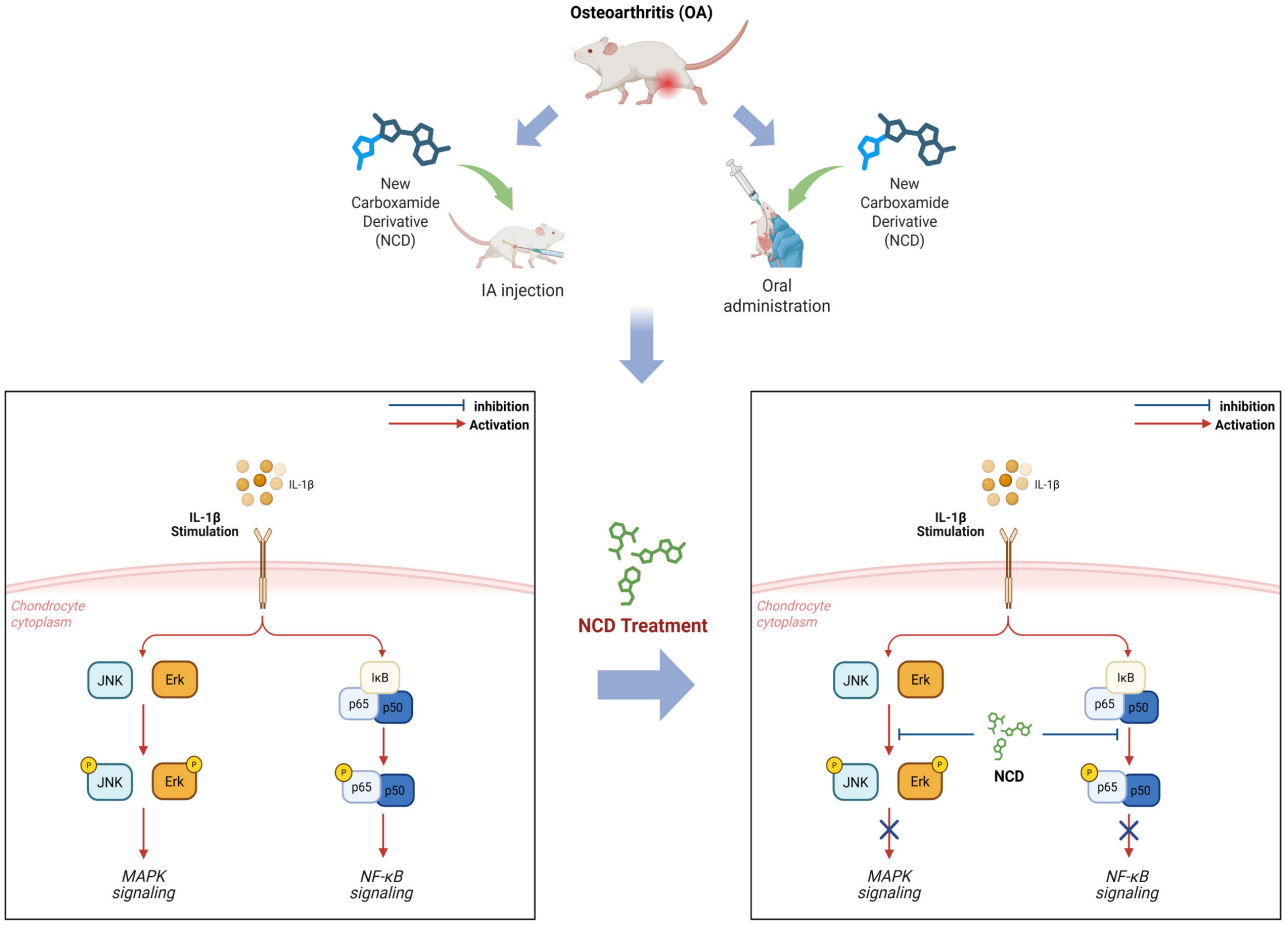
This graphical summary of the study outcomes shows that NCD attenuates OA by suppressing the IL-1β-induced NF-κB and MAPK signaling pathways, with consistent effects observed following both oral administration and IA injection.

To further assess the involvement of the MAPK pathway in NCD-mediated effects, we examined the phosphorylation status of the MAPK subtypes ERK, JNK, and p38 via western blotting and corresponding densitometry. Our results showed that NCD markedly reduced the phosphorylation levels of both ERK and JNK, whereas p38 phosphorylation remained largely unaffected. These findings indicate that NCD selectively inhibits specific MAPK sub-branches while concurrently suppressing the NF-κB signaling cascade. Taken together, these results support the conclusion that NCD mitigates OA responses by epigenetically modulating the transcriptional machinery and by interfering with pro-inflammatory intracellular signaling (**Figure 7**).

## 4 Discussion

OA is associated with various risk factors including aging, joint trauma, and mechanical stress, and leads to the upregulation of multiple pro-inflammatory and catabolic mediators (32). Currently, OA is not curable. Most available treatment methods involve pain control using anti-inflammatory drugs or substances, such as carnosine and hyaluronic acid, through local administration to the joint or surgical process, and help maintain joint function (35). Therefore, we assessed the anti-OA effect of the NCD to establish an effective therapeutic agent for OA.

NCD is a novel Brunauer–Emmett–Teller (BET) inhibitor. Small-molecule inhibitors of BET proteins include I-BET151 and I-BET762 (36). I-BET151 inhibits the expression of inflammatory genes and matrix-degrading enzymes in rheumatoid arthritis synovial fibroblasts. Researchers have focused on its anti-inflammatory cytokine-reducing effects and therapeutic significance in inflammatory diseases (37, 38). JQ1, a well-known BET bromodomain inhibitor, plays a crucial role in regulating arthritis inflammation in bone and joint diseases by blocking IKK-dependent activation of NF-κB signaling in rheumatoid arthritis (39). JQ1 inhibits OA progression by blocking the NF-κB signaling pathway (40); however, JQ1-associated side effects, such as memory and other neurological problems in mice, rapid weight loss, and lymphatic and hematopoietic toxicity at pharmacological concentrations, have been reported (24, 41). We confirmed that cartilage degradation was alleviated by orally administered and injected NCD in DMM mice. Our demonstration of the efficacy of NCD via both oral and intra-articular administration supports its clinical versatility. The dual-route responsiveness observed in this study will expande its therapeutic applicability across both acute and chronic settings, which we hope will provide flexibility for the development of alternative formulations, such as sustained-release oral preparations or long-acting depot injections. This pharmacological adaptability is expected to facilitate patient-centered treatment strategies and enhance the translational potential of this compound. However, as the details of the association between NCD and OA progression remain unknown, we performed additional experiments and obtained favorable results.

In clinical and experimental OA, cartilage destruction and inflammation were caused by increased expression of catabolic factors, such as MMPs and Cox2. Proinflammatory cytokines have been reported to regulate MMPs and Cox2 expression. For instance, IL-1β modulates the expression of catabolic factors in mouse chondrocytes (42, 43). Moreover, it controls downstream molecules by activating the MAPK and NF-κB signaling pathways in chondrocytes and plays a significant role in OA progression (44, 45). Therefore, we used IL-1β to stimulate OA condition *in vitro*.

Mmp3 and Mmp13 exhibit collagenase and aggrecanase activities and accelerate the disruption of ECM components and cartilage injury (15, 16). Prostaglandin synthesis by Cox2 expression is a crucial risk element associated with OA; upregulated prostaglandins improve MMP synthesis (46). In the current study, the NCD suppressed IL-1β-induced OA development in chondrocytes by inhibiting the activity and expression of Mmp3, Mmp13, and Cox2. Hence, we suggest the NCD as a therapeutic agent for OA. Our results indicate that NCD suppressed cartilage destruction via the inhibition of NF-κB and the MAPK signaling pathways.

Mechanistically, BET proteins function as epigenetic readers by recognizing acetylated lysine residues on histone tails, thereby recruiting transcriptional machinery to promote the expression of pro-inflammatory and catabolic genes (47). In chondrocytes, activation of the NF-κB and MAPK pathways enhances histone acetylation at the promoter regions of Mmp3, Mmp13, and Cox2, facilitating BET protein binding and transcriptional activation (21, 48). NCD, as a BET bromodomain inhibitor, likely disrupts this process by competitively binding to acetylated histone sites, thereby preventing the BET protein from being recruited to chromatin and reducing transcriptional elongation of inflammatory genes. This upstream blockade not only suppresses target gene expression directly but also potentially interferes with the feed-forward amplification loop of inflammation, in which activated NF-κB signaling further promotes histone acetylation and chromatin accessibility (27). Here, we observed that NCD treatment led to decreased degradation of IκB and phosphorylation of NF-κB p65, suggesting that BET inhibition affects early signaling events upstream of nuclear translocation. Similarly, the downregulation of phosphorylated ERK and JNK indicates that BET proteins may also facilitate MAPK pathway-dependent gene expression, either directly at the chromatin level or through regulatory feedback loops. These findings support the notion that BET inhibition provides a multi-level blockade of pro-inflammatory signaling cascades in OA pathogenesis.

Our DEL screening identified NCD as a high-affinity binder to BRD2 among BET family members, with favorable TR-FRET and KD values compared to BRD3 and BRD4 (**Figure 2**). These findings demonstrate the molecular selectivity of NCD toward BRD2 among BET bromodomains.

However, while DEL screening provides valuable biochemical evidence of binding specificity, it does not directly demonstrate chromatin-level effects such, as histone acetylation changes or the displacement of BET proteins from gene promoters (49). Therefore, the absence of ChIP assays in this study limits our direct mechanistic insights into how NCD interferes with BET-mediated transcriptional regulation in chondrocytes. Future studies employing ChIP-qPCR or ChIP-seq targeting acetylated histones and BRD2 at the *Mmp3*, *Mmp13*, and *Cox2* promoters will be essential to validate the epigenetic action of NCD in OA.

Furthermore, previous studies have demonstrated that BET proteins act as a shared epigenetic checkpoint, coordinating both the NF-κB and MAPK signaling pathways at the chromatin level. Rather than exerting independent or parallel effects, BET inhibition modulates these pathways through a unified epigenetic mechanism. Specifically, BRD4, a well-characterized BET family member, binds acetylated lysine residues on RelA (p65), promoting the recruitment of BRD4 to NF-κB target gene promoters and facilitating transcriptional activation—a mechanism that has been supported by ChIP and occupancy assays in inflammatory models (50–52). Additionally, BRD4 has been shown to participate in MAPK signaling, with phosphorylation by c-Jun N-terminal kinase (JNK) modulating its chromatin-binding affinity and co-activator function. This crosstalk, demonstrated through ChIP and co-immunoprecipitation analyses, indicates that JNK acts upstream to regulate BRD4-dependent gene expression in stress-responsive contexts (53, 54). Taken together, these findings suggest that BET proteins act as a central chromatin-level integrator of NF-κB and MAPK signaling. Thus, the dual suppression of these pathways by NCD likely reflects inhibition of this shared epigenetic hub. Our current findings provide preliminary support for this model, which we aim to further validate in future ChIP-based studies.

NF-κB is a transcription factor in all animal cells, including chondrocytes. NF-κB plays a crucial role in cellular response to several stimuli, including stress, chemokines, and pro-inflammatory cytokines (55, 56). NF-κB-mediated reactions are initiated by the breakdown of the IκB protein inhibitor bound to NF-κB. After the degradation of IκB, the NF-κB complex is phosphorylated. Subsequently, the phosphorylated NF-κB complex is translocated to the nucleus, and the expression of various mRNA, including MMPs and Cox2, is upregulated. Therefore, induction of the NF-κB signaling pathway causes degradation of articular cartilage and induces OA (32), and OA can be alleviated by suppressing the NF-κB signaling pathway. Based on our results, NCD decreased IκB degradation and p65 phosphorylation; we conclude that NCD can protect against the development of OA by suppressing the NF-κB signaling pathway.

MAPK is a serine/threonine protein kinase associated with signal transduction in eukaryotic cells. Members of the MAPK signaling pathway, including p38, JNK, and ERK, are involved in the development of multiple pathogeneses, including tumor metastasis and certain inflammatory diseases (57–59). Moreover, MAPK signaling significantly influences OA progression (60, 61). The phosphorylation of Erk, Jnk, and p38 is regulated by IL-1β (28). Additionally, the inhibition of ERK signaling helps impaired autophagy to restore OA, and p38 signaling is correlated with the expression of inflammatory factors (62, 63). In the current study, we stimulated articular chondrocytes with IL-1β to induce OA and assessed the change in MAPK signaling after NCD treatment. Our experimental results showed that the NCD suppressed the phosphorylation of Jnk and ERK, but not p38. Hence, we suggest that NCD inhibited the development of arthritis by blocking the MAPK signaling pathway.

While our *in vivo* histological analyses revealed no apparent cytotoxicity in the liver or lungs following both oral and intra-articular administration of NCD (**Supplementary Figures 1** and **Supplementary Figures 2**), we acknowledge that we did not perform a comprehensive safety evaluation including hematological parameters, systemic toxicity profiling, and neurobehavioral assessments. Given that JQ1 has been associated with adverse neurological and hematopoietic effects at therapeutic doses (24, 41), future studies should include these parameters to more thoroughly assess the safety profile of NCD. Such investigations will be critical for fully establishing the safety and translational feasibility of NCD as a disease-modifying therapy for OA.

In conclusion, the NCD, a novel BET inhibitor, suppressed the expression of Mmp3, Mmp13, and Cox2, which caused cartilage destruction, by blocking the NF-κB and MAPK signaling pathways, and it inhibited cartilage degradation in a mouse model of OA. We validated the effects of NCD on OA through *in vitro* and *in vivo* experiments and proposed NCD as a potential candidate for the development of novel drugs that can effectively alleviate OA.

## Data Availability

The datasets presented in this study can be found in online repositories. The names of the repository/repositories and accession number(s) can be found in the article/**Supplementary Material**.
