# Supplementary material for: A novel carboxamide bromodomain inhibitor attenuates osteoarthritis via epigenetic repression of NF-κB and MAPK signaling
Source: Front Immunol. 2025 Jul 31;16:1633334. doi: 10.3389/fimmu.2025.1633334 (PMC12350126; doi:10.3389/fimmu.2025.1633334)
Supplement: Supplementary file 1 [file DataSheet1.pdf]

Supplementary Figures

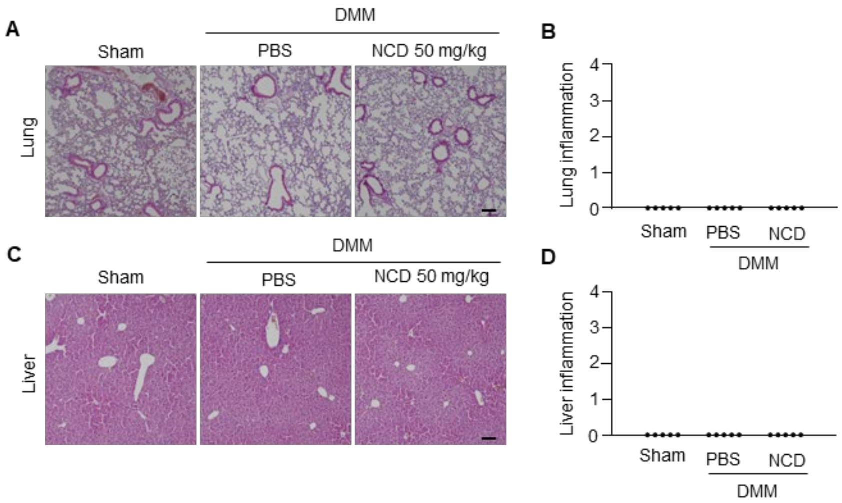

Figure S1. Evaluation of inflammation in lung and liver after NCD oral administration. (A-D) The lungs, and liver of the three groups (sham, DMM, and DMM+NCD 50 mg/kg) were stained to examine histological characteristics (*n* = 5 mice/group) to evaluate the inflammation caused by long-term oral administration of NCD. The images of the lung and liver were captured under the microscope at 100 ×. Scale bar = 100 μm. Sham: control. Values were assessed using the Kruskal-Wallis and Dunn's tests.

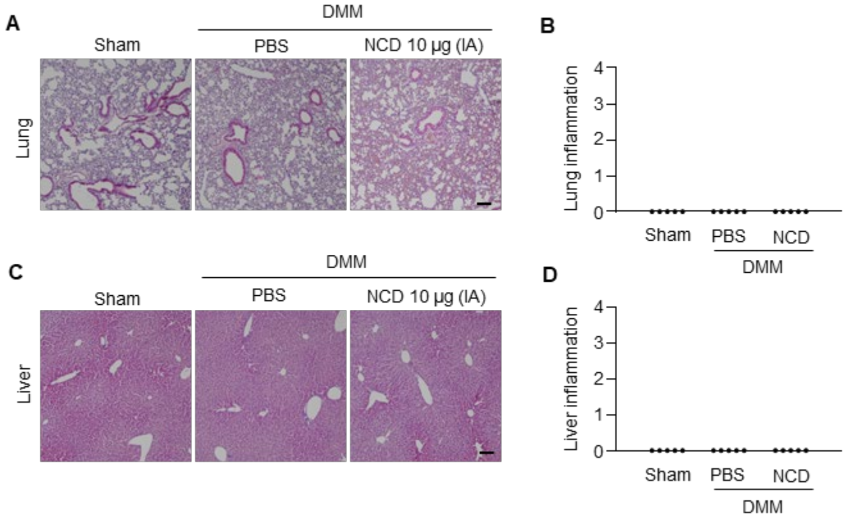

Figure S2. Evaluation of inflammation in lung and liver after intra articular injection of NCD. (A-D) The lungs, and liver of the three groups (sham, DMM, and DMM+NCD 50 µg) were stained to examine histological characteristics (*n* = 5 mice/group) to evaluate the inflammation caused by intra articular injection of NCD. The images of the lung and liver were captured under the microscope at 100 ×. Scale bar = 100 µm. Sham: control. Values were assessed using the Kruskal-Wallis and Dunn's tests. n.s., not significant.

This is a provisional file, not the final typeset article

Supplementary table

Table 1. Pharmacokinetic Parameters of NCD Following Intravenous Administration in SD Rats

NCD	Dose (mg/kg)		
	5	10	20
**Cmax (ng/ml)**	1111.52	2559.25	4150.81
**Half-life (hour)**	0.41	0.59	0.76
**AUC0-t (ng·hr/mL)**	631.83	1728.88	4526.66
